# Enhanced versus standard blood pressure lowering on intracranial aneurysm rupture or growth (ChATIA-1 trial): protocol for a multi-centered, prospective, open-label randomized controlled trial

**DOI:** 10.3389/fneur.2025.1627936

**Published:** 2026-01-07

**Authors:** Kaige Zheng, Shaohua Mo, Zheng Wen, Jiangan Li, Zengli Miao, Xiaojie Lu, Hongwei He, Yong Cao, Bing Zhao, Michael R. Levitt, Lei Chen, Shuo Wang, Chengcheng Zhu, Qingyuan Liu

**Affiliations:** 1 Department of Neurosurgery, Beijing Tiantan Hospital, Capital Medical University, Beijing, China; 2 China National Clinical Research Center for Neurological Diseases, Beijing, China; 3 Department of Neurosurgery, Beijing Xuanwu Hospital, Capital Medical University, Beijing, China; 4 Department of Emergency Medicine, Jiangnan University Medical Center, Wuxi, Jiangsu, China; 5 Department of Neurosurgery, Jiangnan University Medical Center, Wuxi, Jiangsu, China; 6 Department of Neurosurgery, Renji Hospital, Shanghai Jiao Tong University, Shanghai, China; 7 Department of Neurological Surgery, University of Washington, Seattle, WA, United States; 8 Department of Neurosurgery, the First Dongguan Affiliated Hospital, Guangdong Medical University, Dongguan, Guangdong, China; 9 Department of Radiology, University of Washington, Seattle, WA, United States

**Keywords:** unruptured intracranial aneurysm, blood pressure, aneurysm instability, rupture, growth

## Abstract

**Background:**

Hypertension is a common comorbidity in patients with unruptured intracranial aneurysms, which is closely related to the instability of aneurysms. Though anti-hypertension therapy has been recommended by several guidelines, the optimal blood pressure range remains unknown. This trial aims to determine the optimal range of blood pressure by comparing standard blood pressure reduction and intensive blood pressure reduction to reduce the instability of unruptured intracranial aneurysms.

**Methods/design:**

This trial is a multicenter, prospective, open-label, randomized controlled trial with minimization to ensure allocation concealment. Five hundred seventy patients with unruptured intracranial aneurysms and hypertension will be recruited by nine centers in China. Patients will be allocated to the standard blood pressure lowering (SBPL) group (systolic blood pressure at 120–140 mmHg) or the enhanced blood pressure lowering (EBPL) group (systolic blood pressure at <120 mmHg). The primary outcome is aneurysm instability within 24 months after appropriate blood pressure lowering therapy, including aneurysm growth and aneurysm rupture. During follow-up, Blood pressure data will be collected monthly, while radiological examination for aneurysms will be performed at 6 ± 1 months, 12 ± 1 months and 24 ± 1 months. The trial has 85% power to reduce 60% of aneurysm instability.

**Discussion:**

The China Antihypertensive Trial for Intracranial Aneurysm (ChATIA) trial is a randomized trial about blood pressure lowering therapy for UIA patients. In this study, a new strategy and evidence will be provided for blood pressure management in the future.

**Clinical trial registration:**

http://www.clinicaltrials.gov, identifier NCT05941377.

## Introduction

Unruptured intracranial aneurysms (UIAs) are pathological dilatations of cerebral arteries that develop under the combined effects of hemodynamic stress and progressive vascular wall degeneration (1, 2). A comprehensive systematic review and meta-analysis encompassing 68 studies from 21 countries (83 cohorts, 94,912 individuals) estimated the global prevalence of UIAs in adults around 50 years of age to be approximately 3.2% (3). In China, a cross-sectional study of 4,813 Shanghai residents aged 35–75 years reported a higher prevalence of 7.0%, peaking in individuals aged 55–64 years (4). Given the catastrophic consequences of aneurysmal rupture and the procedural risks associated with prophylactic surgical or endovascular repair, optimizing medical management to prevent aneurysm growth or rupture has emerged as a critical unmet clinical need (1, 5).

Hypertension is the most prevalent comorbidity in this population (1, 6). Its influence on aneurysm instability—manifested as aneurysm growth or rupture—is profound (7). Hypertension is a well-established, independent risk factor for rupture, primarily through its augmentation of hemodynamic stress on the fragile, degenerative aneurysmal wall. This excessive pressure accelerates mural fatigue, promotes inflammatory remodeling, and ultimately compromises wall integrity (8). Consequently, hypertension is incorporated into validated rupture risk models such as the PHASES score, and its presence markedly increases rupture probability, even in small aneurysms (≤7 mm) (9). Given the central role of hypertension, strict blood pressure control constitutes a cornerstone of conservative UIA management (10, 11).

In the broader cardiovascular literature, randomized controlled trials have demonstrated the benefits of intensive blood pressure reduction. The SPRINT trial showed that targeting a systolic BP < 120 mmHg significantly decreased major cardiovascular events (12). Similarly, the STEP trial (target 110–130 mmHg) reduced major cardiovascular events, including stroke, while the BPROAD trial found that intensive BP lowering reduced such events in patients with diabetes (13, 14).

However, definitive randomized evidence specific to UIA patients remains lacking. The safety and efficacy of intensive blood pressure targets for preventing aneurysm growth or rupture have not been rigorously tested, leaving the optimal management threshold unresolved.

To address this gap, we initiated the China Antihypertensive Trial for Intracranial Aneurysm (ChATIA)—a randomized controlled study designed to evaluate the benefits and safety of enhanced blood pressure reduction in patients with unruptured intracranial aneurysms. This trial aims to establish evidence-based guidelines for blood pressure management tailored to the unique pathophysiological context of UIAs.

## Methods/design

### Study design

The ChATIA trial is a multicenter, prospective, open-label, randomized controlled trial with a minimization system to allocate patients with UIAs into the SBPL group and EBPL group at a ratio of 1:1. The primary efficacy endpoint is UIA instability, and the safety endpoint is the incident of ischemic cerebral or cardiac events. This randomized controlled trial (RCT) will follow the Consolidated Standards of Reporting Trials (CONSORT) guidelines (15).

### Patient selection

The inclusive/exclusive criteria are listed as follows:

#### Inclusion criteria

1. Age 18–75.
2. Chinese ethnicity.
3. Unruptured saccular intracranial aneurysm (UIA) identified by computational tomography angiography, magnetic resonance angiography or digital subtraction angiography.
  - Maximal size of UIA at largest dimension < 7 mm
  - The morphology of UIA is regular (no bleb(s) or secondary aneurysm(s) protruding from the UIA fundus or bi−/multi-lobular UIA fundus)
  - UIA receiving conservative treatment
4. History of primary hypertension (as diagnosed per standard of care)
5. Systolic blood pressure (SBP) on 2 consecutive visits:
  SBP: 130–180 mmHg on 0 or 1 antihypertensive medication.
  SBP: 130–170 mmHg on up to 2 antihypertensive medications.
  SBP: 130–160 mmHg on up to 3 antihypertensive medications.
  SBP: 130–150 mmHg on up to 4 antihypertensive medications
6. Good medication adherence (Morisky Medication Adherence Scale ≥6)
7. Obtain informed consent from patient or legal representative.

##### Exclusion criteria

1. Patients with neurological symptom related to UIA, such as sentinel headache, oculomotor paralysis and so on.
2. Patients with additional active intracranial disease including vasculopathy, arteriovenous malformation/fistula, cancer, traumatic brain injury etc.
3. Patients with fusiform, dissecting, blister, traumatic, mycotic/bacterial, myxomatous, and tumor-associated UIAs are excluded.
4. Patients with history of polycystic kidney disease, rheumatic disease or autoimmune disease.
5. Patients with family history of intracranial aneurysm (defined when two direct relatives of patients within three generations have intracranial aneurysms or aneurysmal subarachnoid hemorrhage).
6. Patients with known secondary cause of hypertension.
7. Patients with myocardial infarction, ischemic stroke, symptomatic heart failure during the past 3 months.
8. Patients with a medical condition likely to limit survival to less than 2 years.
9. Patients during pregnancy and perinatal period.
10. Any concurrent serious illness that would interfere with the outcome assessments including hepatic, renal, gastroenterological, respiratory, cardiovascular, endocrinologic, immunologic, and hematologic disease.
11. Inability or unwillingness of patient or legal representative to give written informed consent.
12. Participation in a concurrent interventional medical investigation or clinical trial.

UIA patients who meet the inclusion criteria but do not meet the exclusion criteria will be considered in subsequent randomization.

### Outcomes and relative definitions

The primary efficacy outcome is UIA instability, including aneurysm growth, rupture or aneurysm-related symptoms during 24-month follow-up. The definitions of UIA instability are listed as follows:

1. Aneurysm growth is defined as one or more of the following: (1) growth of more than 1 mm in at least one direction, (2) growth of more than 0.5 mm in at least two directions, (3) growth of more than 20% of baseline aneurysm size in any direction, or (4) new appearance of irregularity on the UIA.
2. Aneurysm rupture is diagnosed when patients have the symptoms of non-traumatic subarachnoid hemorrhage (e.g., severe headache and sudden coma) attributable to the target UIA, imaging evidence of hemorrhage on CT or MRI, and/or bloody cerebrospinal fluid from lumbar puncture.
3. Occurrence of aneurysm-related symptoms is diagnosed as sentinel headache (a sudden and severe headache attributable to the UIA within 2 weeks of onset, and without prior history of significant headache within the previous 5 years), oculomotor nerve palsy (a sudden headache with one or several symptoms of pupillary light reflex disappearing, ptosis, or extraocular myoparalysis, ipsilateral to a UIA in the vicinity of the affected oculomotor nerve and without systemic cause such as diabetes), other cranial neuropathy (such as visual field defect secondary to UIA compression of the optic apparatus without alternative cause), or seizure related to UIA inflammation (defined as EEG or clinically determined seizure activity generated from inflammatory changes in the brain parenchyma surrounding the UIA on imaging, and without prior history of seizure or other cause of seizure).

Two investigators who are unaware of the patient’s information assess the occurrence of UIA instability and ischemic brain or cardiac events at 6 months (± 1 month), 12 (± 1 month) months, and 24 (± 1 month) months through monthly follow-up and imaging examinations of UIA.

The primary safety outcome is any ischemic cerebral or cardiac events during 24-month follow-up. This includes one or more of the following: new or more frequent transient ischemic attacks, new clinical or radiological ischemic stroke, angina, new myocardial infarction, or reperfusion therapy for myocardial infarction. Ischemic brain or cardiac events are defined as follows:

1. TIA is defined as a transient episode (duration of symptoms < 24 h) of neurological dysfunctions caused by focal brain, spinal cord, or retinal ischemia without showing acute infarction on non-contrasted CT (NCCT).
2. Ischemic stroke is a persistent episode of neurological dysfunctions caused by focal brain, spinal cord, or retinal ischemia, which shows acute hypodense infarction area on NCCT images and T2-weighted MRI.
3. Angina is defined as a transient chest pain or pressure (duration of symptoms < 30 min) caused by insufficient blood flow to the myocardium without significant ST/T changes in ECG and rise of cardiac biomarkers.
4. Myocardial infarction is defined as myocardial necrosis in clinical settings consistent with myocardial ischemia. These conditions can be satisfied by a rise of cardiac biomarkers [preferably cardiac troponin (cTn)] plus at least one of the following: (1) symptoms of cardiovascular ischemia; (2) ECG changes indicative of new ischemia (significant ST/T changes or left bundle branch block); (3) development of pathologic Q waves; (4) imaging evidence of new loss of myocardium or new regional wall motion abnormalities; (5) angiography or autopsy evidence of intracoronary thrombus.

Secondary outcomes are: 1. UIA rupture events during 24-month follow-up; 2. UIA growth events during 24-month follow-up; 3. Change in average Wall Enhancement Index (WEI) at baseline and 24th month. WEI is defined as follows:

Two investigators (with work experience of >10 years, who are blind to the clinical and morphological information) measure the WEI value independently using Vessel-MASS software (Leiden University Medical Center). Three slices with significant enhancement observed by post-contrast VW-MRI at baseline and 24 months will be selected. After tracing the lumen and outer boundaries of the aneurysm wall, the contours of the inner lumen and outer wall of aneurysms will be automatically segmented using the software. If the tracking is not satisfactory, the investigators will manually adjust the contour of the inner cavity and the outer wall. Subsequently, the mean signal intensity (SI) will be calculated automatically using the software. The average and maximum value of the mean SI of the corresponding three slices will be used for further analyses.


$$\mathrm{WEI}=\frac{\frac{\mathrm{SI}\_\mathrm{Wal}l_{\text{postcontrast}}}{\mathrm{SI}\_\text{White matte}r_{\text{postcontrast}}}-\frac{\mathrm{SI}\_\mathrm{Wal}l_{\text{precontrast}}}{\mathrm{SI}\_\text{White matte}r_{\text{precontrast}}}}{\frac{\mathrm{SI}\_\mathrm{Wal}l_{\text{precontrast}}}{\mathrm{SI}\_\text{White matte}r_{\text{precontrast}}}}$$

### Consent

The patients, or their legal representatives, are approached by the principal investigator or sub-investigator of the clinical team to ascertain their interest in participating in the ChATIA trial. When patients or their legal representatives freely and voluntarily agree to participate in this study, their signature or name/seal and the date of consent shall be obtained based on a consent form approved by the participating hospitals.

### Procedure and data collection

Data collection includes demographic information, clinical information, and radiological information. The details are shown in Table 1.

**Table 1:** Study procedures.

ChATIA-1 trial	Study procedures				
	Screening	Follow-up			
	Outpatient visit	During 24-month follow-up	6 ± 1 months	12 ± 1 months	24 ± 1 months
Randomization	○				
Clinical information	○				
Radiological examinations for UIA	○		○	○	○
Monthly Blood pressure monitor		○			
Aneurysmal symptom monitor		○			
Ischemic event monitor		○			

UIA, unruptured intracranial aneurysm.

**Figure 1:**
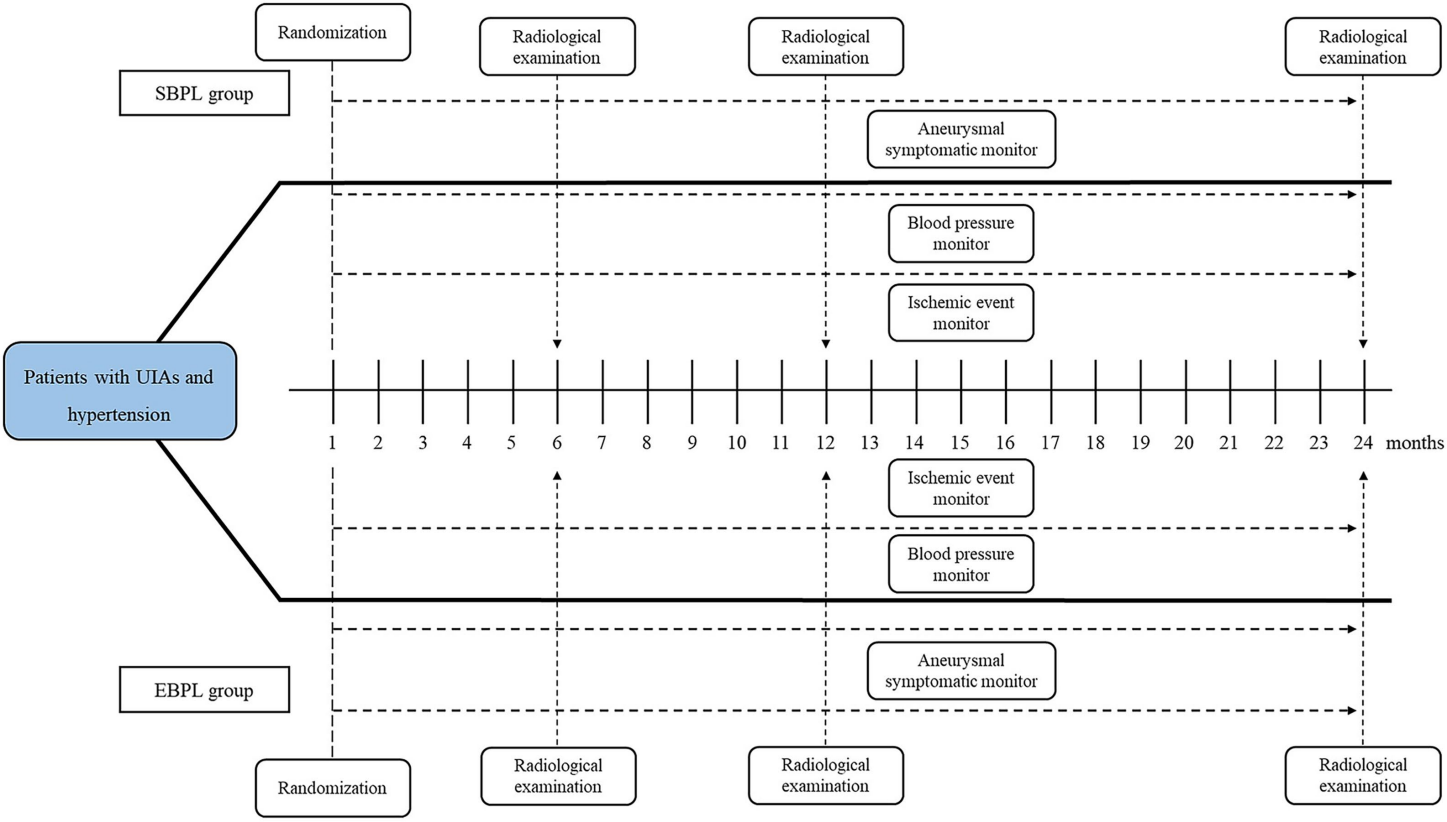
The flow chart of study procedure. UIA, unruptured intracranial aneurysm; SBPL, standard blood pressure lowering; EBPL, enhanced blood pressure lowering.

The procedure includes patient screening and enrollment (Step 1), randomization and intervention (Step 2), and outcome evaluation (Step 3), as shown in Figure 1.

#### Step 1: Screening and enrollment

The patients, or their legal representatives, are approached by the principal investigator or sub-investigator of the clinical team to ascertain their interest in participating in the ChATIA trial. Potential participants with UIAs will primarily be identified and recruited from outpatient clinics, where eligible patients are regularly followed for aneurysm management and blood pressure control. When patients or their legal representatives freely and voluntarily agree to participate in this study, their signature or name/seal and the date of consent shall be obtained based on a consent form approved by the participating hospitals.

Patients will receive a primary screening after obtaining consent. The evaluation includes radiological examination for UIA [CT angiography (CTA) and vessel wall (VW)-MRI], blood pressure in the past month, exclusionary conditions and medication adherence.

#### Step 2: Randomization and intervention

Enrolled UIA patients will be randomized at a ratio of 1:1 to the EBPL group and SBPL group after obtaining informed consent. Dynamic randomization using the minimization method is used as the randomization of subjects to the EBPL or SBPL group. Randomization and enrollment of subjects are made using the online randomization system. Randomization factors are as follows: age at the time of obtaining the informed consent (≥ 60 years or < 60 years), sex (female or male), aneurysm location [Anterior Communicating Artery (Acom)/Anterior Cerebral Artery (ACA), Internal Carotid Artery (ICA), Middle Cerebral Artery (MCA) and Posterior circulation (PC)]. Patients in the EBPL group are required to keep systolic blood pressure at <120 mmHg and those in the SBPL group at 120-140 mmHg.

This study aims to compare the effects of different blood pressure targets rather than specific medications. Therefore, the blood pressure treatment protocol is flexible in terms of the choice and doses of antihypertensive medications, but there should be preferences based on cardiovascular outcome trials results and current guidelines [such as thiazide diuretic, Calcium Channel Blockers (CCBs), Angiotensin-Converting Enzyme Inhibitors (ACEIs), and Angiotensin II Receptor Blockers (ARBs)]. If adopting another dose frequencies is not required (e.g., twice a day), the preference will be choosing long-acting antihypertensive agents, i.e., once per day.

The cardiologists may select among the available study antihypertensive medications for initiation of therapy. Other drugs not supplied by the trial may also be used as the cardiologists determines appropriate. However, all antihypertensive regimens should include one or more drug classes with strong cardiovascular outcome data from large randomized controlled hypertension trials. If three drugs are needed, a thiazide-type diuretic, a RAS blocker (ACEI or ARB, but usually not both), and CCB make a very effective and usually well-tolerated regimen. The decision is left to the investigator as long as the BP goals are achieved.

**Figure 2:**
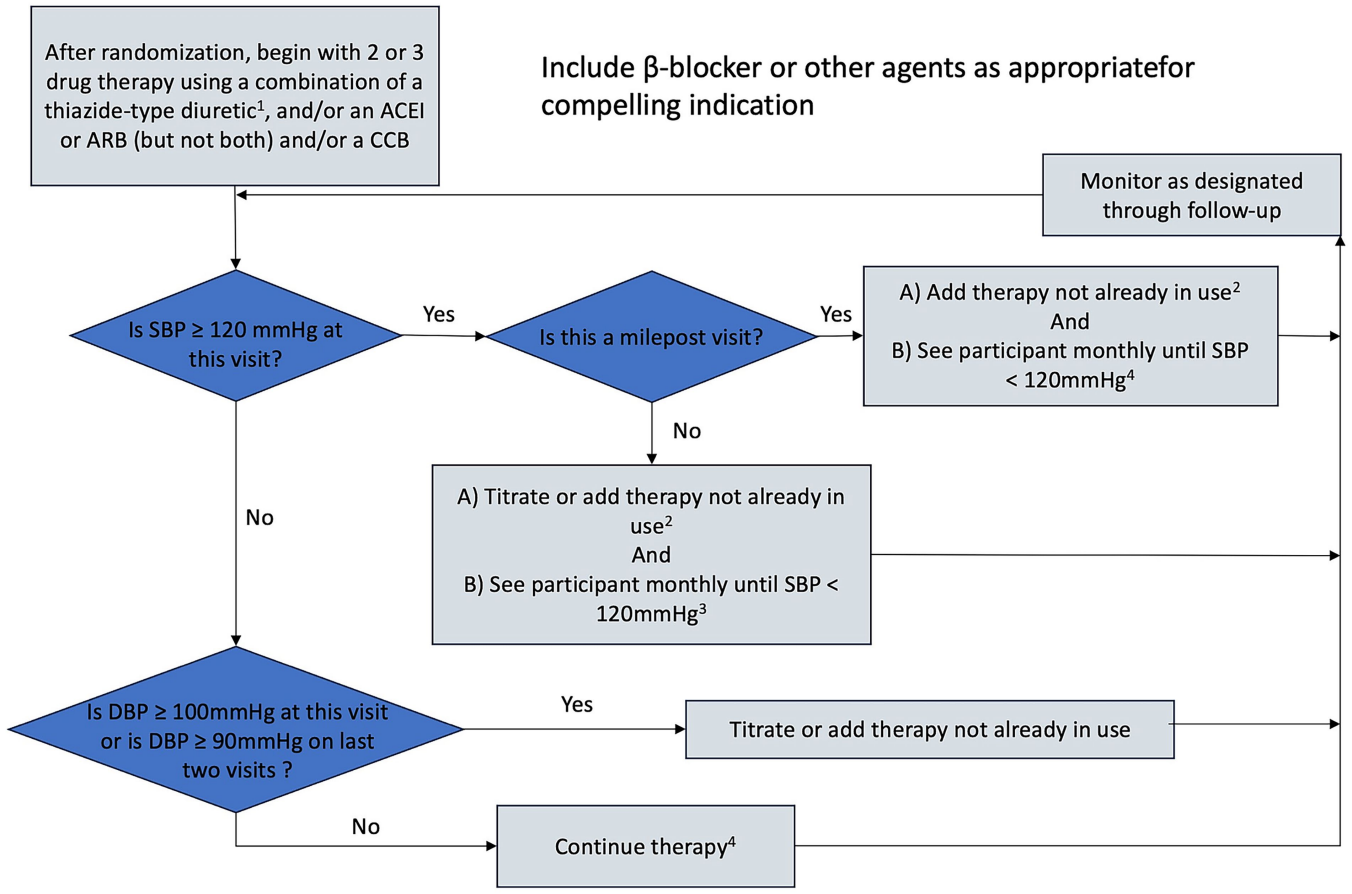
Treatment algorithm for EBPL group (SBP < 120 mmHg). 1. May use loop diuretic for patients with advanced CKD. 2. Consider consulting a cardiologist before adding a fifth antihypertensive medication, 3. Or until clinical decision made that therapy should not be increased further. 4. Unless side effects warrant change in therapy.

**Figure 3:**
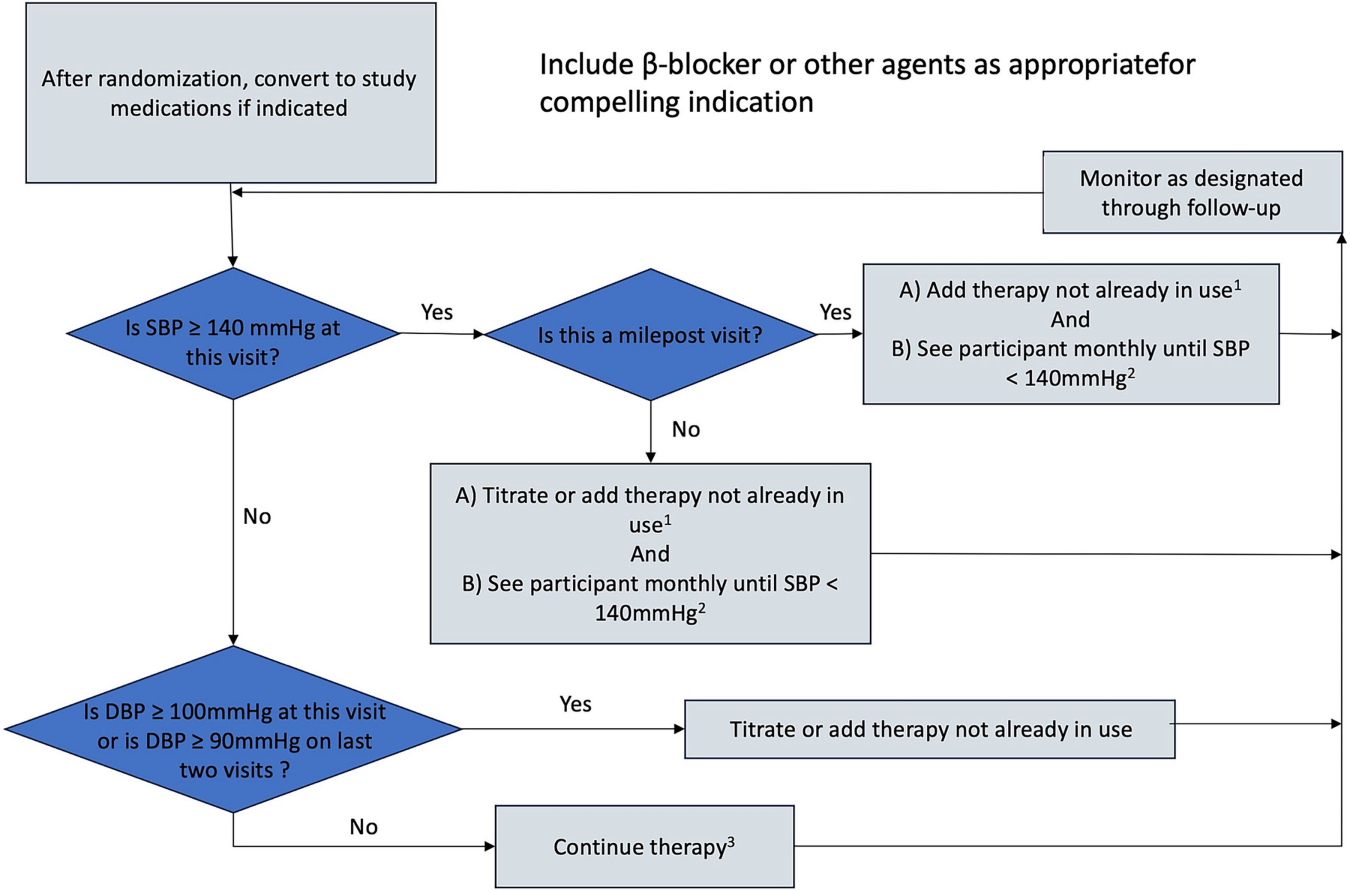
Treatment algorithm for SBPL group (SBP at 120–140 mmHg). 1. Consider consulting a cardiologist before adding a fifth antihypertensive medication, 2. Or until clinical decision made that therapy should not be increased further. 3. Unless side effects warrant change in therapy.

Once the SBP goal has been achieved in any participant, the antihypertensive regimen should be intensified if diastolic blood pressure (DBP) remains ≥100 mmHg at a single visit or ≥90 mmHg at two successive visits to achieve DBP < 90 mm Hg. The visit intervals and decisions for titration (other than the BP levels) will be similar to those used for the SBP goal. Since beta-blockers reduce DBP more than systolic BP relative to other antihypertensive medications, a beta-blocker could be considered for such participants (Figures 2, 3).

#### Step 3: Outcome evaluation

All patients will be required to complete head CTA at 6 months (± 1 month), 12 (± 1 month) months, and 24 (± 1 month) months during follow-up. VW-MRI examinations are required to be completed at 24 (± 1 month) months after appropriate blood pressure lowering therapy.

Self-record blood pressure and occurrence of any aneurysm-related symptoms or safety endpoints (ischemic cerebral or cardiac events) will be recorded every month via outpatient or phone review during follow-up.

Patients will use self-monitoring methods to monitor blood pressure during the follow-up. All patients are required to measure the blood pressure of the left brachial artery twice per week. Before blood pressure measurement, patients will be required to rest quietly for at least 5 min. Blood pressure will be measured using the YUYUE electronic sphygmomanometer (YE660A, China, which can record 40 data points in built-in memory). The data on blood pressure will be collected once a month (8-week blood pressure data will be collected from an electronic sphygmomanometer). The average of 8 systolic pressures will be calculated and used as the monthly pressure in statistical analysis.

For both randomized groups, protocol visit frequency will be monthly. During follow-up, outcome assessors who are blinded to patients’ treatment allocation will independently record all relevant events in detail:

1. UIA instability (the occurrence time, treatment and radiological data);
2. Monthly self-monitoring blood pressure (8-week blood pressure data will be collected from an electronic sphygmomanometer. The average of 8 systolic pressures will be calculated and used as the monthly pressure);
3. Ischemic cerebral or cardiac events (the occurrence time, treatment and radiological data).
4. Regular follow-up head CTA [6 months (± 1 month) 12 (± 1 month) months, and 24 (± 1 month) months].

Milepost visit: Milepost visits are used to assist in reaching goal SBP. In this trial, milepost visits will be every 6 months throughout follow-up in both the EBPL group and SBPL group (6th month, 12th month, and 18th month). During each milepost visit, patients will be asked to visit the nearest participating hospital, where cardiologists will measure their blood pressure. This measured blood pressure will be recorded as the milepost visit blood pressure. If the SBP exceeds the target value at milepost visit, then an antihypertensive drug from a class different from what is being taken should be added. Once participant has been prescribed five drugs at maximally tolerated doses, if the SBP remains above goal at subsequent milepost visits, it will be permitted to substitute a different class into the regimen instead of adding another drug or increasing the dose of a drug. However, additional (more than 5) drugs may be needed to achieve goal SBP in some participants. Medication adherence will be assessed routinely in the current trial and should be evaluated especially carefully for participants not at goal on 4 or more medications. Strategies to enhance adherence should be applied.

### Quality control of the research

To avoid bias and ensure patients’ medical adherence, we follow the standards in the subsequent text:

1. Self-monitoring of blood pressure: According to the recommendation of China’s guideline for hypertension (2018) (16), we will use the home blood pressure monitoring method to monitor patients’ blood pressure monthly. All patients will receive training for blood pressure measurements from professional cardiologists at enrollment and every 3 months. We will evaluate pre-enrollment medication adherence and include only patients with good adherence. We will require all patients to measure the blood pressure of the left brachial artery twice at least 1 min apart in the morning before breakfast and taking antihypertensive medications, and after urination and defecation. Before blood pressure measurement, all patients will be required to rest for at least 5 min. Blood pressure is measured using the YUYUE electronic sphygmomanometer (YE660A, China). Researchers will collect blood pressure data once a month.
2. Protocol-noncompliance: If the patient’s SBP does not reach the assigned target during follow-up, the cardiologists will evaluate the patient according to the predefined antihypertensive treatment algorithm (Figures 2, 3).
3. Unable to complete the required CTA examinations: We will contact each patient one month before the CTA examination by telephone to ensure completion. If patients are unable to complete CTA examinations in the required time interval, they will not be included in the final analysis.

### Safety monitoring

Each participating investigator has primary responsibility for the safety of the individual participants under his/her care. At each follow-up, CRC will specifically query participants for adverse events (AE) and serious adverse event (SAE) and recorded them in the case report form (CRF). Event Adjudication Committee members are responsible to make final ascertain of safety outcomes.

In our current study, adverse event (AE) is defined as any intervention related unfavorable medical outcomes, including any clinically obvious symptom, abnormal sign, or disease. In our current study, we will monitor AE including hypotension, dizziness, angioedema, headache.

Adverse event is considered as serious adverse event (SAE) only when it met following criteria: fatal or life-threatening, result in significant or persistent disability, or require hospitalization or surgical intervention, including:

- Symptomatic hypotension.
- Arrhythmia.
- Acute kidney failure.
- Electrolyte abnormalities.
- Injurious falls.
- Syncope.
- Unexpected events for which the investigator believes that intervention caused the event or contributed to the immediate cause of the event.

### Statistical analysis

#### Sample size

The sample size is estimated using the PASS 15 (PASS Corporation, United States). In our preliminary studies, the 2-year instability events risk was approximately 20.7% in UIA patients with hypertension and 11.5% in UIA patients with controlled hypertension (17). In this study, we assume that EBPL could decrease the risk of aneurysm rupture and growth by 50%. At *α* = 0.05, *β* = 0.90, we need to enroll 506 patients (253patients in the SBPL group and 253 patients in EBPL group). By estimating the clinical dropout rate at 10%, the required sample size was calculated to be 282 patients in each group. This study prepared to include 570 patients in both groups (285 in each group), which could meet the statistical requirements.

#### Endpoint and outcome evaluation

Before locking the database for analysis, we intend to test its plausibility and effectiveness. We will exclude the cases that could influence the accuracy of the conclusion. Patients who do not meet the requirement of quality control may be further excluded based on the consensus of the investigators’ discussion.

Categorical variables are presented as numbers (no.) and percentages (%). Continuous variables with normal distribution are expressed as means and standard deviation, and medians and inter-quartile range (IQR) if otherwise. The differences between groups in continuous variables are compared by performing the student’s t-tests or Wilcoxon rank sum tests, and the differences in categorical variables are compared using the chi-square tests or Fisher’s exact tests. The incident rate (IR) of PR and its 95% confidence interval (CI) are calculated. The univariate and multivariate Cox regression analyses are performed in PR-related analysis. The result of Cox regression analysis is presented as hazard ratio (HR) and 95% CI. SPSS 24.0 (SPSS, Chicago, IL) is used for statistical analysis, and bilateral *p* < 0.05 is considered statistically significant.

### Subgroups analysis

Five subgroups are analyzed, including the aneurysm location subgroup, the aneurysm maximum diameter subgroup, the different antihypertensive regimen subgroup, the aspirin use subgroup and the statin use subgroup. Subgroup analyses are limited to the primary efficacy endpoint and safety endpoint only. In the subgroup analysis, a separate Cox model will be applied for each subgroup.

### Data management

The clinical research coordinator (CRC) will conduct regular visits to each participating center to ensure the strict implementation of the research program. If there is any conflict with the research program, CRC will be reported to investigators in a timely manner. In addition, throughout this study, a summary meeting will be held every 3 months to discuss and solve any problem arising from the research or encountered by the patients.

All data will be collected and stored using a case report form (CRF), and the completed CRFs will be sent back per 3 months. In addition, the data will be also transcribed into an electronic version by nine investigators independently. After completion, the CRFs will be sealed in a research-specific cabinet and electronic data will be stored by a specific person. A data management committee was established to supervise data quality and ensure data security. Data is only open to research team members. The privacy of patients is protected.

### Ethical considerations

This study was approved by the Institutional Review Board. The research would be conducted in strict accordance with the declaration of Helsinki and Human Biomedical Research Ethical Issues. All enrolled subjects will be informed with a complete and comprehensive introduction, including the study’s purpose, procedure, potential risks, and potential benefits. The patient’s family should sign the written informed consent before beginning. They will be informed that they have the right to withdraw from the study at any time, and informed consent will be retained as a clinical study document for future reference. The subject’s personal privacy and data confidentiality will be protected during the study.

### Participating institutions

Beijing Tiantan Hospital, Capital Medical University.

Beijing Friendship Hospital, Capital Medical University.

Beijing Chaoyang Hospital, Capital Medical University.

Beijing Anzhen Hospital, Capital Medical University.

Renji Hospital, Shanghai Jiao Tong University.

Jiangnan University Medical Center.

First Affiliated Hospital of Fujian Medical University.

Guangzhou Red Cross Hospital, Jinan University.

Binzhou Medical University Hospital.

## Discussion

Hypertension has long been considered a risk factor for aneurysm growth and rupture (1, 11). The ChATIA study is the first clinical trial to investigate the optimal blood pressure management of Chinese UIA patients. In this study, we investigate whether enhanced blood pressure lowering therapy can prevent UIA instability without increasing the risk of ischemic cerebral or cardiac events.

The formation, growth and rupture of the intracranial aneurysm have been proven to have a strong correlation with hypertension. Abnormal hemodynamics and destruction of arterial walls induced by hypertension play a significant part in intracranial aneurysm growth and rupture (18, 19). Previous studies and guidelines recommend effective antihypertensive therapy can reduce the risk of aneurysm instability (17, 20). Notably, intensive blood pressure reduction therapy may increase the risk of ischemic cerebral and cardiac events as reported in several studies (12–14). However, the optimal blood pressure range for UIA patients remains unknown.

The ChATIA trial aims to investigate the optimal blood pressure management strategy for the Chinese UIA population. The efficacy and safety of enhanced antihypertensive therapy will be further verified in this study.

## Conclusion

In summary, we have described a clinical trial for enhanced blood pressure reduction therapy for UIA patients with hypertension. The efficacy and safety of blood pressure reduction will be discussed in this study, which will provide evidence for blood pressure management in the future.
